# Supplementary material for: Three year clinical outcomes in a nationwide, observational, siteless clinical trial of atrial fibrillation screening—mHealth Screening to Prevent Strokes (mSToPS)
Source: PLoS One. 2021 Oct 5;16(10):e0258276. doi: 10.1371/journal.pone.0258276 (PMC8491919; doi:10.1371/journal.pone.0258276)
Supplement: Attachment — Submitted filename: Revision Response to Editor & Reviewer.docx [file pone.0258276.s003.docx]

**Response to Editors and Reviewer:**

(Reviewer comments in black and *our responses in blue*.)

**Comments for the Editor:**

1. Please ensure that your manuscript meets PLOS ONE's style requirements, including those for file naming.

Changes have been made to be in compliance with all style requirements.

2. Please include your amended Competing Interests Statement within your cover letter.

The amended Competing Interests Statement has been included in the cover letter.

3. Please remove any funding-related text from the manuscript and let us know how you would like to update your Funding Statement.

All funding-related text has been removed from the manuscript.

It seemed to us that the Funding Statement is accurate as written and no changes were made to that.

4. Please upload a copy of Supporting Information Table which you refer to in your text on page 4.

The Supplementary Table of Diagnostic codes used to identify each clinical outcome has been uploaded.

5. Please review your reference list to ensure that it is complete and correct.

All references have been reviewed and corrected as needed

**Reviewer #1:**

1. Abstract: it should be added how screening for AF was performed.

The first sentence in the Methods section of the abstract was modified to say the following:

*“…to either immediate or delayed screening using a continuous-recording ECG patch to be worn for two weeks and 2 occasions, ~3 months apart, in order to potentially detect undiagnosed AF.”*

2. 42.9% of patients hospitalized for AF: this appears high and should be commented

We agree with the Reviewer and were surprised by this, although we don’t believe that the hospitalization was specifically “for” just AF as ~27% experienced a stroke, heart failure, heart attack or systemic emboli. As far as we can tell, there is no other literature describing the occurrence of clinical events surrounding the new diagnosis of AF. We added some additional discussion around this finding in the Conclusion of the Abstract and in the Discussion section.

3. Methods. Sample size calculation should be better performed. Quotations of incidence of AF should be added

We have modified the section describing the sample size calculation and have added references for the basis of our estimations. One added reference was our study design manuscript which goes into more detail of our sample size calculations. The modified sentence is;

*“Based on internal health plan data and available incidence data we anticipated an incidence of AF of 10% in both cohorts during the 3-year follow-up and an expected incidence of the combined endpoint of 12% in the control cohort and 5% among the actively monitored [15, 16],…”*
